# Exploring the Bioactive Potential and Chemical Profile of *Schinus molle* Essential Oil: An Integrated *In Silico* and *In Vitro* Evaluation

**DOI:** 10.3390/plants14152449

**Published:** 2025-08-07

**Authors:** Rómulo Oses, Matías Ferrando, Flavia Bruna, Patricio Retamales, Myriam Navarro, Katia Fernández, Waleska Vera, María José Larrazábal, Iván Neira, Adrián Paredes, Manuel Osorio, Osvaldo Yáñez, Martina Jacobs, Jessica Bravo

**Affiliations:** 1 Centro Regional de Investigación y Desarrollo Sustentable de Atacama (CRIDESAT), Universidad de Atacama, Av. Copayapu Nr 485, Copiapó 1530000, Chile; romulo.oses@uda.cl; 2 Laboratorio de Hormonas y Biología del Cáncer, Instituto de Medicina y Biología Experimental de Cuyo (IMBECU), CONICET CCT-Mendoza UNcuyo, Mendoza CP 5500, Argentinaflabruna@gmail.com (F.B.); 3 Laboratorio de Productos Bioactivos, Facultad de Medicina, Centro de Investigación Biomédica, Universidad Diego Portales, Ejército 141, Santiago 8370007, Chile; patricio.retamales@mail.udp.cl (P.R.); myriam.navarro@mail.udp.cl (M.N.); katia.fernandez@udp.cl (K.F.); manuel.osorio@unab.cl (M.O.); martinajacobs@ug.uchile.cl (M.J.); 4 Facultad de Salud y Odontología, Universidad Diego Portales, Ejército 278, Santiago 8370007, Chile; 5 Laboratorio de Química de Metabolitos Bioactivos, Facultad de Farmacia, Escuela de Química y Farmacia, Universidad de Valparaíso, Avenida Gran Bretaña 1095, Valparaíso 2360102, Chile; waleska.vera@uv.cl; 6 Centro de Investigación, Desarrollo e Innovación de Productos Bioactivos, CInBIO, Facultad de Farmacia, Universidad de Valparaíso, Avenida Gran Bretaña 1095, Valparaíso 2340000, Chile; 7 Departamento de Ciencias de los Alimentos y Nutrición, FACSA, Universidad de Antofagasta, Angamos 601, Antofagasta 1240000, Chile; maria.larrazabal@uantof.cl; 8 Departamento de Tecnología Médica, FACSA, Universidad de Antofagasta, Angamos 601, Antofagasta 1240000, Chile; ivan.neira@uantof.cl; 9 Laboratorio Química Biológica, Instituto Antofagasta and Departamento de Química, Universidad de Antofagasta, Angamos 601, Antofagasta 1240000, Chile; adrian.paredes@uantof.cl; 10 Facultad de Odontología, Universidad Andrés Bello, Echaurren 237, Santiago 8370133, Chile; 11 Centro de Modelación Ambiental y Dinámica de Sistemas (CEMADIS), Facultad de Ingeniería y Negocios, Universidad de Las Américas, Santiago 7500975, Chile; 12 Núcleo de Investigación en Data Science, Facultad de Ingeniería y Negocios, Universidad de las Américas, Santiago 7500975, Chile

**Keywords:** essential oil, *Schinus molle*, antioxidant, *Caenorhabditis elegans*, antiproliferative, toxicity, antimicrobial activity, chemical composition

## Abstract

Chilean *Schinus molle* has been used in traditional medicine for effects such as antibacterial, antifungal, anti-inflammatory, analgesic, antiviral, antitumoral, antioxidant, antispasmodic, astringent, antipyretic, cicatrizant, cytotoxic, diuretic, among others. In this study, we evaluated the pharmacological potential of *Schinus molle* seed essential oil extract (SM_EO) through *in vitro* and *in silico* approaches. *In vitro*, the antioxidant potential was analyzed, and antitumor activity was evaluated in non-tumor and human epithelial tumor cell lines. *Caenorhabditis elegans* was used as a model for evaluating toxicity, and the chemical composition of the SM_EO was analyzed using gas chromatography–mass spectrometry. The oil contained four major monoterpenes: α-phellandrene (34%), β-myrcene (23%), limonene (13%), and β-phellandrene (7%). Based on quantum mechanical calculations, the reactivity of the molecules present in the SM_EO was estimated. The results indicated that α- phellandrene, β-phellandrene, and β-myrcene showed the highest nucleophilic activity. In addition, the compounds following these as candidates for antioxidant and antiproliferative activities were α-phellandrene, β-phellandrene, ρ-cymene, sabinene, caryophyllene, l-limonene, and α-pinene, highlighting *β*-myrcene. Based on ADME-Tox properties, it is feasible to use these compounds as new drug candidates. Moreover, the antibacterial activity MIC value obtained for *B. cereus* was equivalent to 2 μg/mL, and for *Y. enterocolitica*, *S. enteritidis*, and *S. typhimurium*, the MIC value was 32.5 μg/μL. SM_EO could selectively inhibit the proliferation of human epithelial mammary tumor MCF7 cells treated with SM_EOs at 64 and 16 ug/mL—a significant increase in BCL-2 in a dose-dependent manner—and showed low toxicity against *Caenorhabditis elegans* (from 10 to 0.078 mg·mL^−1^). These findings suggest that SM_EO may be a potential source of bioactive compounds, encouraging further investigation for applications in veterinary medicine, cosmetics, and sanitation.

## 1. Introduction

*Schinus molle* L. is a large plant of the Anacardiaceae family, native to the American tropics, which can reach up to 8 m in height under wilderness conditions [1]. Due to its resistance to pollution, this plant is often used ornamentally; however, it also has a long history of applications in traditional medicine worldwide [2]. Its popular uses include the treatment of diseases such as pediculosis, mitigating rheumatism and sciatica symptoms, and pest control. The above is attributed to the antibacterial, antifungal, antiseptic, analgesic, healing, and anti-inflammatory properties of *Schinus molle* aerial parts [3]. The tree bears reddish fruits that are used in traditional cuisine as a replacement for black pepper due to their spiciness and in the preparation of alcoholic brews [1,4]. Chilean *Schinus molle* is locally known for its antibacterial, antifungal, healing, and antispasmodic effects. Leaf preparations are commonly used to attenuate stomach and liver ailments, rheumatic pain, and menstrual irregularities [5]. However, no studies have been found that scientifically validate the properties of the variety found in Chile, nor of the oil extracted from the seeds.

Essential oils (EOs) are complex mixtures of volatile organic compounds produced by subjecting the plant material to distillation processes [6]. Plants produce these precursor EO compounds as secondary metabolites, frequently involved in defensive mechanisms [7]. EO chemical composition varies even when extracted from the same species; research suggests that the chemotypes are influenced by the plant structure from which the oil is extracted (leaves, berries, wood, etc.), as well as the location and season of harvest [8]. In the case of *Schinus molle* essential oil (SM_EO), although characterizations of the leaf EO predominate, those focused on the fruits reveal notoriously different results [9]. A total of 57 compounds were found in the SM_EO from berries collected in Tunisia [8], with α-phellandrene (46.52%), β-phellandrene (20.81%), and α-terpineol (8.38%) found to be predominant. Martins [10] characterized the fruit SM_EO from trees in Portugal, finding a higher proportion of β-myrcene (51.3%), limonene (14.1%), and α-phellandrene (14.0%), with a total number of 16 different compounds. Hosni [11] studied the influence of the fruit-ripening stage on the composition of the EO, finding predominant concentrations of α-phellandrene (35.15–40.38%), limonene + β-phellandrene (21.47–36.62%), and β-myrcene (7.61–24.96%) in immature, intermediate, and mature stages, respectively. Giuffrida [12] carried out a characterization of the compounds in the fruit pulp SM_EO to assess its use in traditional cuisine, finding 46 compounds in the volatile fraction, with sabinene (51.74%), limonene (16.98%), and terpinen-4-ol (4.93%) found to be predominant among the monoterpenes, and germacrene B (5.12%) among the sesquiterpenes. Different C10 monoterpenes and C15 sesquiterpenes are present in the aroma profile. Limonene, linalool, and β-pinene are present in strawberries (*Fragaria x ananassa*), koubo (*Cereus peruvianus* L.), and citrus (*Citrus* sp.) [13,14,15]. The contribution of each compound to the specific volatile profile depends on each crop, with the variety and the physiological behavior of the fruit predominating. It also depends on the activity and specificity of the enzymes involved in the biosynthetic pathways and the odor threshold above which the compound can be detected in the presence of others [16]. The greatest variability in volatile composition occurs in fresh fruit, affecting the fruit’s genetic makeup and ripeness. The abundance of these volatiles in fruits is related to cultivation, ripeness, and harvest handling [17]. The highest volatile content is observed in ripe fruit. Unripe fruit is easy to store and transport, but it lacks flavor due to the direct relationship between ripeness and volatile biosynthesis [18].

Given that SM_EO is a complex mixture that may contain over 50 compounds, an *in silico* analysis of its main components can provide relevant information about each individual’s contribution. Quantum mechanical calculations have been used to estimate the reactivity of organic compounds of pharmacological interest based on reactivity indicators, charge distribution, and antioxidant power [19,20]. These indices predict the likelihood of participation in nucleophilic/electrophilic attacks or generation of free radicals, which are molecular reactivity determinants. In addition to evaluating their capacity to carry out chemical reactions, it is crucial to determine the toxicity of the compounds of interest by identifying interactions with metabolism or transport [21]. These factors can be assessed through prediction programs that analyze physicochemical properties and interactions with enzymes involved in metabolic detoxification or transport processes. These analyses are used to assess the viability of new drugs as well as to identify the pharmacological potential of new compounds.

In terms of antioxidant potential, [22] evaluated the essential oil from leaves collected in Uruguayan, Brazil, employing the methods of DPPH, ABTS, and FRAP, and observed a low antioxidant capacity. The antioxidant activity showed an IC50 of 36.3 µg mL^−1^ [23].

Although recent studies support the antibacterial and antifungal capacity of *Schinus molle* leaf EO and extracts [24,25], only a few studies have tested the berry’s antimicrobial effects. Martins and collaborators evaluated the antimicrobial activity of essential oils (EOs) from berries and leaves using disc diffusion and minimal inhibitory concentration (MIC) assays [10]. Berry EOs were effective against *Staphylococcus aureus* and *Enterococcus faecalis*, and both EOs showed efficacy against antibiotic-resistant Gram-negative bacteria, such as *Pseudomonas aeruginosa* and *Salmonella Typhimurium*. MIC tests revealed that berry EO had activity against Gram-positive strains, particularly *Staphylococcus epidermidis* and *Staphylococcus aureus*, but was less effective than leaf EO. Eryigit and collaborators found that berry EO exhibited enhanced inhibitory activity against *Bacillus subtilis*, *Enterococcus faecalis*, and *Saccharomyces cerevisiae*, and showed a modest inhibitory effect of 8 mm against *Escherichia coli* and *Salmonella Typhimurium* [26].

The essential oil was shown to be cytotoxic in several cell lines, demonstrating that it is more effective on breast carcinoma and leukemic cell lines. The LD50 for cytotoxicity at 48 h in K562 corresponded to 78.7 µg mL^−1^, which was very similar to the LD obtained when apoptosis was measured [23]. The essential oil did not induce significant necrosis up to 200 µg mL^−1^, which, together with the former results, indicates that apoptosis is the main mechanism of toxicity induced by SM_EO in this cell line [23]. Duarte [27] evaluated the cytotoxicity and genotoxicity in human lymphocyte and macrophage cultures. The leaf essential oil presented cytotoxic effects against both cell types, and genotoxicity was only detected in macrophages and *Caenorhabditis elegans* [28].

Therefore, this study aims to characterize the chemical profile and bioactive potential of *Schinus molle* seed essential oil (SM_EO), integrating *in vitro* and *in silico* approaches, in order to provide a foundational understanding of its pharmacological relevance and guide future translational applications.

## 2. Materials and Methods

### 2.1. Collection, Identification, and Extraction

*Schinus molle* seeds were collected at the beginning of the flowering season in February 2020, 5 km to the north of the sector called “Travesia” (27°30′00″ S 70°25′00″ W), Copiapó, at 750 m.a.s.l., Region of Atacama, Chile (the aerial parts). The identified plant corresponds to a specimen deposited in the Herbarium of the Faculty of Natural and Oceanographic Sciences, Department of Botany, University of Concepcion (accession No 125,734 CONC). A volume of 1 L of essential oil was obtained from 35 kg of fresh seed via classical steam distillation for 2 h at atmospheric pressure in pilot-scale stainless-steel equipment [29,30]. The hydrolase was subjected to centrifugal separation to recover and combine the oily material with the oil obtained. The steam distillation yielded 7%. Anhydrous sodium sulfate was used to dry the extract, which was stored at 4 °C in darkness until further analysis.

### 2.2. Essential Oil Analysis (GC-MS)

The chemical profiling of the extract was conducted via gas chromatography–mass spectrometry (GC-MS) analysis using a Shimadzu QP 2010 Plus system equipped with an Rtx-5MS fused silica capillary column (30 m × 0.25 mm ID; film thickness: 0.25 µm) operating in splitless injection mode and coupled to a Shimadzu mass detector. The analytical conditions were as follows: Helium served as the carrier gas at a constant flow rate of 1.0 mL/min, with an injection volume of 1.0 µL. Mass spectra were recorded in full-scan mode across a range of 35–500 *m*/*z* at a scan frequency of 1.56 scans/s. Electron impact ionization (EI+) was employed at 70 eV, with the ion source maintained at 280 °C. The temperature program was initiated at 50 °C for 5 min, followed by a ramp of 8 °C/min up to 280 °C, where it was held for 15 min. The terpenes present in the essential oil were identified by comparing their retention indices (RI) with a homologous series of n-alkanes (C10–C30; Supelco) analyzed under identical experimental conditions. The standards, procured from Sigma^®^ and confirmed to have 99% purity, were co-injected for validation. Compound identification relied on spectral matching against the NIST 05 and Wiley MS libraries (NIST/EPA/NIH Mass Spectral Library with Search Program, version 2.0 g; NIST data version 11) accessible online at http://www.nist.gov/srd/nist1a.cfm and comparison with previously reported MS data (accessed on 4 January 2024) [31].

### 2.3. Computational Methods

#### 2.3.1. Density Functional Theory (DFT) Calculation

Functional density geometric optimizations were performed for compounds exceeding 1% abundance: α-pinene, β-caryophyllene, α-phellandrene, p-cymene, β-myrcene, β-phellandrene, sabinene, and l-limonene (isomer of higher reactivity) from the SM_EO from Chile. Together, these 8 compounds represented 94.34% of the total mixture. To evaluate the individual contribution to the antioxidant capacity, antimicrobial and antiproliferative activity of the mixture, for each of these 8 molecules, DFT calculations were performed with PBE0-D3(BJ)/Def2-TZVP level using the Gaussian16 software suite [32,33]. Water was included in the simulations as a solvent with SMD parametrization of the IEF-PCM [32]. The vibrational frequencies were computed to verify that the optimized structures are minima in their corresponding potential energy surface. All three-dimensional structures of the compounds were downloaded from the PubChem database (https://pubchem.ncbi.nlm.nih.gov/, accessed on 17 July 2025) [33,34,35,36,37,38,39,40,41,42], using the Chemical Abstracts Service (CAS) identifier. This unique identifier ensures precise compound selection, guaranteeing data reliability for subsequent analysis. Using DFT calculations, we determined the global reactivity descriptors (HOMO–LUMO gap, ionization potential, electron affinity, electronegativity, global hardness, electrophilicity indices ω, ω^−^, ω^+^, and Δω±) and local descriptors (Fukui functions f^+^, f^0^, f^−^), which provide critical insights into electronic stability, reactive sites, and molecular behavior, enabling comprehensive characterization of their functional properties (Table S1).

#### 2.3.2. Molecular Electrostatic Potential (MEP) Surfaces

Furthermore, for a comprehensive analysis of SM_EO molecules, we generated three-dimensional MEP surfaces. These surfaces visually illustrate the distribution of electronic charges within the molecules, offering valuable insights into their chemical reactivity and site-specific interactions. By employing color-coded MEPs, which represent variations in electron density, we established a predictive framework for electrostatic interaction potentials. Regions exhibiting positive potentials (depicted in shades of blue) are identified as potential targets for nucleophilic attacks. In contrast, those with negative potentials (transitioning from green to red) denote areas of electrophilic character, thus predisposing them to nucleophilic interactions. This comprehensive approach enhances our understanding of molecular behavior. It provides essential information for further analysis and interpretation [43].

#### 2.3.3. Pharmacokinetics and Toxicity

All pharmacokinetics and toxicity calculations were performed on the ADMETlab 2.0 web server [44]. ADMETlab 2.0 is an online platform that provides comprehensive and accurate predictions of absorption, distribution, metabolism, excretion, and toxicity (ADMET) properties for chemical compounds. It supports the prediction of 88 ADMET-related endpoints, including 17 physicochemical properties, 13 medicinal chemistry properties, 23 ADME properties, 27 toxicity endpoints, and 8 toxicophore rules (751 substructures). ADMETlab 2.0 employs a multi-task graph attention (MGA) framework to develop robust and accurate prediction models. The MGA framework uses graph neural networks and self-attention mechanisms to learn representations of molecular graphs and capture long-range dependencies between atoms. In addition, all models from the ADMETlab 2.0 web server were trained using the random forest algorithm and optimized hyperparameters [44].

### 2.4. Antioxidant Capacity

#### 2.4.1. Chemical Materials

The reagents used in the antioxidant assays were procured from reliable suppliers. Specifically, 1,1-diphenyl-2-picrylhydrazyl radical (DPPH), 2,4,6-tris-(2-pyridyl)-s-triazine (TPTZ), (-)-6-hydroxy-2,5,7,8-tetramethylchromane-2-carboxylic acid (Trolox), potassium persulfate (K_2_S_2_O_8_), ferrozine, HPLC-grade water, and sodium phosphate salts (monobasic NaH_2_PO_4_ and dibasic Na_2_HPO_4_) were obtained from Sigma-Aldrich (St. Louis, MO, USA). Additional reagents, including iron (III) chloride hexahydrate (FeCl_3_·6H_2_O), iron (II) sulfate heptahydrate (FeSO_4_·7H_2_O), hydrochloric acid (HCl), methanol (MeOH), 2,2′-azino-bis(3-ethylbenzothiazoline-6-sulfonic acid) diammonium salt (ABTS), acetic acid, sodium acetate trihydrate (CH_3_COOH·3H_2_O), and disodium EDTA, were sourced from Merck (Darmstadt, Germany).

#### 2.4.2. FRAP Assay

The FRAP assay was performed using a previously described method [45]. Briefly, 10 μL of the SM_EO solution at a final concentration of 500 μg·mL^−1^ (10 mg EO dissolved in 1 mL absolute EtOH and subsequently diluted to obtain a working solution of 500 μg·mL^−1^) [45,46] was mixed with 70 μL of freshly prepared FRAP solution at a ratio of 10:1 (*v*/*v*) [300 mM acetate buffer (pH 3.6), 10 mM 2,4,6-tris-(2-pyridyl)-s-triazine (TPTZ) dissolved in 40 mM HCl, and 20 mM FeCl_3_·6H_2_O aqueous solution]. After incubating the solution for 30 min at 37 °C, absorbance was measured at 593 nm with a microplate reader (BioTek Synergy HTX Multimodal Kit; Winooski, VT, USA), and the values were interpolated onto a Trolox calibration curve over a concentration range of 0–500 μg·mL^−1^. The FRAP results were expressed as milligrams of Trolox equivalents per mL of SM_EO. All experiments were performed in triplicate.

#### 2.4.3. Metal Chelating Activity (Ferrozine)

The chelating activity of SM_EO against iron (II) ions was assayed using the method described by [47] with some modifications. Briefly, 50 μL of ΕO at a final concentration of 500 μg·mL^−1^ was mixed with 10 μL of 2 mM FeCl_2_ (prepared in HPLC-grade water). The reaction was started by the addition of 20 µL of 5 mM ferrozine (prepared in HPLC-grade water). The total volume was adjusted to 150 µL with methanol (70 µL). After incubation at 25 °C for 10 min, absorbance was measured in triplicate at 562 nm with a microplate reader (BioTek Synergy HTX Multimodal Kit). EDTA (0–250 μg·mL^−1^) was used as the standard control. The results were expressed as milligrams of EDTA equivalents per mL of SM_EO.

#### 2.4.4. DPPH Radical Scavenging Assay

The free radical scavenging activity of SM_EO was quantified using previously described methodology [48]. A 70 μL aliquot of 0.2 mM DPPH solution, prepared in methanol, was mixed with 20 μL of essential oil (EO) or Trolox at concentrations ranging from 0 to 1000 μg·mL^−1^. The mixture was incubated in the dark at room temperature (22 °C) for 30 min. Following incubation, the absorbance was measured using a BioTek Synergy HTX multimodal microplate reader. All experiments were performed in triplicate, and the results were reported as IC_50_ values (μg·mL^−1^), defined as the EO concentration required to scavenge 50% of the DPPH radicals in the solution.

#### 2.4.5. ABTS Radical Scavenging Assay

The ABTS radical scavenging activity of SM_EO was quantified following a previously described method [48]. The working ABTS solution was prepared by mixing 7 mM ABTS in PBS (pH 7.4) with 2.5 mM K_2_S_2_O_8_ (final concentration), followed by storage in the dark at room temperature for 16 h to allow radical generation. Afterward, the mixture was diluted with PBS to achieve an absorbance of 0.700 ± 0.02 units at 734 nm. Subsequently, 20 μL of each diluted solution of SM_EO or Trolox (used as the standard) within the concentration range of 0–1000 μg·mL^−1^ was combined with 180 μL of the ABTS solution. Absorbance measurements were taken 6 min after the initial mixing at 734 nm using a BioTek Synergy HTX multimodal microplate reader. All experiments were conducted in triplicate.

The scavenging activity was reported as IC_50_ values (μg·mL^−1^), which represent the concentration of SM_EO required to neutralize 50% of the ABTS radicals in the solution. A blank control consisting of 180 μL of ABTS solution and 20 μL of PBS was used for baseline correction.

### 2.5. Antibacterial Activity

#### 2.5.1. Chemical Materials and Antibiotics

The stocks of antibiotics used, including ampicillin (10 mg·mL^−1^ and 100 mg·mL^−1^) and ciprofloxacin (1 mg·mL^−1^), as well as monoterpenes α-phellandrene and limonene (99% pure), were purchased from Sigma-Aldrich (St. Louis, MO, USA).

#### 2.5.2. Microbial Strains

The bacterial strains used for microbiological tests included the reference strains *Escherichia coli* ATCC 35218, *Enterococcus faecalis* ATCC 51289, *Listeria monocytogenes* ATCC BAA 751, *Pseudomonas aeruginosa* ATCC 1744, *Staphylococcus aureus* ATCC 25923, *Salmonella enteritidis* ATCC 13076, *Staphylococcus epidermidis* ATCC 16624, *Staphylococcus sciuri* ATCC 29061, *Salmonella typhimurium* ATCC 14,028, and *Yersinia enterocolitica* ATCC 9338361, and clinical isolates of *Bacillus cereus*, *Corynebacterium striatum*, *Shigella boydii* 39, *Shigella flexneri* 44, *Salmonella Paratyphi* B, and *Shigella sonnei*. The bacterial strains were obtained from the Microbiology Laboratory collection, Department of Medical Technology, Universidad Diego Portales, kindly provided by Pedro Cortés. The strains were grown on Mueller Hinton Agar at 37 °C [49,50].

#### 2.5.3. Microplate Assay

To determine the minimum inhibitory concentration (MIC), the microdilution technique was performed in 96-well plates following the guidelines established by the Clinical and Laboratory Standards Institute (CLSI). Serial 10-fold dilutions of SM_EO were prepared in Mueller Hinton BD^®^ broth and distributed across the wells of the 96-well plates. Fresh bacterial suspensions, adjusted to a density equivalent to 1 × 10^8^ CFU·mL^−1^, were used for inoculation. The plates were subsequently incubated under conditions previously specified in the CLSI protocol [51].

SM_EO was dissolved in 2% (*v*/*v*) DMSO, a concentration that does not interfere with bacterial growth, and added to the inoculated wells. Following incubation for 24 h, absorbance readings at 550 nm were recorded to determine the MIC, as outlined in the CLSI guidelines. Control wells without SM_EO and wells without bacterial inoculation served as negative controls, while positive controls included antibiotics to ensure bacterial sensitivity. Statistical analyses were conducted using ANOVA in GraphPad Prism 5 to assess significant differences between experimental groups [51].

### 2.6. Cytotoxicity

#### 2.6.1. Cell Line Culture

To evaluate the selective cytotoxicity of the SM_EO on human epithelial non-tumor and tumor cells lines, epithelial mammary cells MCF10A (non-tumoral) and MCF7 (tumoral) and epithelial renal cell lines HK-2 (non-tumoral), 786-O, and ACHN (tumoral) were obtained from the American Type Culture Collection (ATCC, Rockville, MD, USA). The cells were cultured in Dulbecco’s modified Eagle’s F-12 medium (DMEM-F12; Gibco, Waltham, MA, USA) supplemented with 10% fetal bovine serum (FBS; Gibco, Waltham, MA, USA) and maintained at 37 °C under 5% CO_2_. When the cells reached 80% confluence, the cultures were replicated. The numbers of cell passages were approximately 5–10. *In vitro* assays were performed with triplicates of three independent experiments for each cell line [21].

#### 2.6.2. Crystal Violet Proliferation Assay

To investigate the impact of SM_EO on human epithelial tumor cells, a dose–response analysis was conducted across specified concentrations, along with a time-course evaluation. Cells were initially detached using 0.5% Trypsin-EDTA (Gibco, Waltham, MA, USA), and 5 × 10^3^ cells per well were seeded into 96-well plates containing DMEM-F12 medium supplemented with 10% FBS. The plates were incubated for 24 h to allow cell adhesion. After incubation, the supernatant was removed, and cells were washed once with 1× PBS (Gibco, Waltham, MA, USA). Subsequently, cells were treated with increasing concentrations of SM_EO (64, 32, 16, 8, 4, or 2 µg·mL^−1^) prepared in DMEM-F12 supplemented with 1% FBS. The negative control group received a vehicle treatment consisting of 1% DMSO in DMEM-F12 with 1% FBS, which was also used to dissolve SM_EO.

After 24 or 48 h of treatment, the supernatant was discarded, and cells were washed with 1× PBS. A 100 µL volume of crystal violet (CV) staining solution (0.2% *w*/*v* in 10% ethanol; Gibco, Waltham, MA, USA) was added and incubated for 20 min. The CV solution was then removed, and the intracellular dye was eluted with 0.1 M Na_2_HPO_4_ (pH 4.5, in a 50:50 *v*/*v* ethanol solution; Sigma, St. Louis, MO, USA). Absorbance readings were taken at 570 nm for each sample. The results were expressed as the percentage of color intensity relative to the control group and normalized to the values of cells treated with 1% DMSO alone [21,48].

#### 2.6.3. Western Blot Analysis

The RIPA buffer was used to lyse MCF7 cells and Micro BCA (Protein Assay Kit, Thermo Scientific Inc., Waltham, MA, USA) to quantify the total proteins in samples. Proteins were separated in an SDS-PAGE 13% gel and electro-transferred to a PVDF membrane. Afterward, the membranes were washed with TBS-T 0.1% buffer 1X (Tris-base 20 mM, NaCl 137 mM, 0.1% Tween-20) and blocked with 5% (*w*/*v*) human serum albumin (Sigma-Aldrich, 0055 K, St. Louis, MO, USA). The membranes were washed with TBS-T 0.1% and incubated overnight at 4 °C with the following primary antibodies: BCL-2 (ab4051, 1:300 dilution, Abcam plc), P53 (ab32138, 1:500 dilution, Abcam plc), RB and β-actin (sc-47778, 1:1000 dilution, Santa Cruz Biotechnology Inc., Dallas, TX, USA). Afterward, membranes were incubated for 90 min at room temperature in the presence of the horseradish peroxidase-conjugated secondary antibody (1:3000 polyclonal goat anti-rabbit biotinylated, Dako Cytomation, Glostrup, Denmark). After five washes in TBS-T, specific bands were detected via chemiluminescence (ECLTM, Amersham, Sigma Aldrich, Dorset, UK) using a ChemiDoc XRS+ System with Image Lab Software (Version 6.1) from Bio-Rad (Hercules, CA, USA). To achieve consistent quantification across Western blot bands, the narrowest band was selected as a reference to standardize an area, which was then systematically applied to all bands. Only the pixel intensity in this standardized area was quantified, ensuring a uniform area size throughout the analysis following the guidelines outlined in the FIJI Image Processing package guide (x86-64). In cell extracts, β-actin levels were utilized as a loading control to verify equal protein loading in the gel.

Independent data were analyzed using one-way ANOVA, followed by Tukey’s post hoc test. *p* < 0.05 was considered to be statistically significant [21,48].

### 2.7. Toxicity

#### 2.7.1. Maintenance of Caenorhabditis Elegans Culture

For toxicity assays, the wild strain of *Caenorhabditis elegans*, N_2_, was used. The N_2_ strain was maintained on agar plates with nematode growth medium (NGM) in the presence of a layer of *Escherichia coli* OP50. These plates were incubated at 20 °C for 3 days. Subsequently, gravid nematodes were collected and treated in the presence of a chlorine solution (0.45N NaOH and 2% HOCl) to obtain eggs. To hatch the eggs and obtain synchronized adult nematodes, eggs were placed in plates with OP50 for 3 days. Finally, nematodes were collected in M9 saline solution (1.5 g KH_2_PO_4_, 3 g Na_2_HPO_4_, 2.5 g NaCl, 0.5 mL of 1M MgSO_4_, and distilled water to raise the final volume to 500 mL) [52].

#### 2.7.2. Test Preparation

SM_EO was prepared at concentrations of 0.078, 0.156, 0.31, 0.62, 1.25, 2.5, 5, and 10 mg·mL^−1^ and added to 96-well plates at a final volume of 100 µL per well. Negative control treatments included 1% DMSO and M9 saline solution. Each well was inoculated with 10 adult *Caenorhabditis elegans* nematodes. The plates were incubated at 20 °C for 24 and 48 h. All experiments were conducted in triplicate and repeated twice.

To assess the survival rate, all nematodes were examined after 24 and 48 h. Nematodes were classified as alive if they exhibited any movement of the tail, head, or pharynx during a 5 s observation period; otherwise, they were considered dead. The mortality rates were calculated based on the observed counts. [21,48].

## 3. Results

### 3.1. Composition

**Figure 1:**
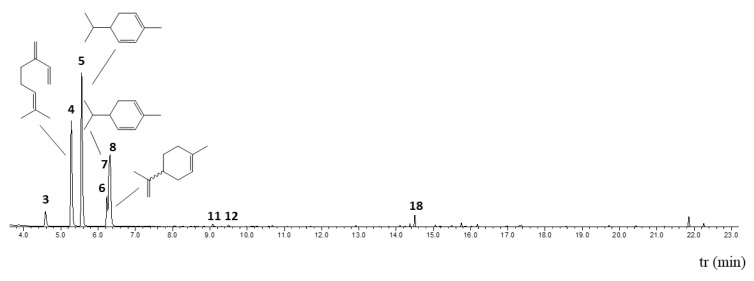
Chromatography profile of SM_EO. The peaks observed correspond to the major chemical compounds, mainly monoterpenes.

SM_EO samples were analyzed via GC-MS (Table 1, Figure 1: tr, retention time; I.R^.e^, retention or relative Kovats index calculated relative to a C10-C30 n-alkane standard on an Rtx-5MS capillary column; I.R.^t^, retention or relative Kovats index reported in the literature; IRNR, retention or relative Kovats index not reported; Area%, percent area under the curve of each peak indicating the amount of product in the total sample; Id, identification of the chemical compound present; and CAS, Chemical Abstracts Service number). The match (>75) was performed by comparing the mass spectra with those of NIST 14, together with co-elution with standard compounds available in the laboratory. The colored quadrants correspond to products in higher proportion (α-phellandrene 34.02%, β-myrcene 23.87%, limonene 13.99%, and β-phellandrene 7.01%).

### 3.2. Reactivity of SM_EO Components

HOMO–LUMO analysis, fundamental in quantum chemistry, serves as a powerful tool elucidating the intricate movement of electrons within molecules, including those inherent in SM_EO. By delving into the energy levels of the HOMO and LUMO ($\epsilon_{L})$, this analysis unveils not only the chemical reactivity and stability but also the intricate details regarding electronic transitions and charge transfer phenomena within these molecules [53]. A narrower HOMO–LUMO gap facilitates electron transfer, thereby increasing the reactivity of the molecule. Moreover, the distribution of the frontier molecular orbitals provides insights into the regions of the molecule that exhibit the highest reactivity [53]. On the other hand, a higher energy level of the HOMO correlates with an increased ability to engage in nucleophilic attacks, which is a significant electronic parameter for evaluating the antioxidant capacity of a compound [54]. Antioxidants function by donating electrons to neutralize free radicals, thereby preventing oxidative damage. A higher HOMO energy level indicates that the molecule can more readily donate electrons, enhancing its effectiveness as an antioxidant. This is because a higher HOMO energy means that less energy is required to remove an electron from the HOMO, making the molecule a better electron donor [54]. This property is essential in the development of new antioxidant compounds, where the goal is to maximize the molecule’s electron-donating capacity to improve its protective effects against oxidative stress. Such insights extend beyond mere stability assessments; they offer a nuanced understanding of reactive sites, electron-donating/accepting propensities, and the broader reactivity landscape of SM_EO constituents.

#### 3.2.1. Frontier Orbitals

The HOMO–LUMO analysis (see Table S1) of several compounds found in SM_EO provides insightful data on their electronic properties and potential reactivity. Notably, *α*-phellandrene exhibits the smallest HOMO–LUMO gap (GAP) of 5.46 eV, indicating a higher reactivity due to easier electron transfer. This is followed closely by *β*-myrcene and *β*-phellandrene, with gaps of 5.64 and 5.77 eV, respectively, suggesting they too are relatively reactive. On the other hand, *α*-pinene and *l*-limonene display larger gaps of 6.91 and 6.85 eV, respectively, implying lower reactivity. The HOMO energies ($\epsilon_{H})$ also vary, with *α*-phellandrene having the highest HOMO energy (−6.04 eV), suggesting it might be the most efficient in nucleophilic attacks, a valuable trait for antioxidant activity. Conversely, *ρ*-cymene has the lowest HOMO energy (−6.59 eV), potentially making it less reactive in nucleophilic processes.

Koopman’s theorem-based DFT calculations of global (IP, EA, χ, η, ω, ω±, Δω±) and local descriptors for eight major *S. molle* components revealed β-myrcene as the most electrophilic (ω = 2.18 eV, Δω± = 5.06 eV) due to optimal electron acceptance (EA = 0.68 eV) and donation (ω− = 4.28 eV) capacity, while ρ-cymene showed greater stability (η = 3.21 eV) but strong electronegativity (χ = 3.39 eV), collectively explaining their distinct reactivity patterns for pharmacological applications; for more details, see Supplementary Materials (Table S2).

These reactivity indices (IP, EA, ω) directly correlate with bioactive properties: lower IP enhances antioxidant potential through improved electron donation, while higher EA facilitates electron acceptance; reduced electrophilicity (ω) further minimizes unwanted cellular interactions, optimizing therapeutic suitability [55]. *β*-myrcene emerges as the most promising compound due to its favorable combination of descriptors, including a relatively low *IP* of 6.32 eV, a high *EA* of 0.68 eV, and a moderate *ω* of 2.18 eV, indicating strong antioxidant potential and balanced reactivity [56]. *β*-phellandrene and *α*-phellandrene follow closely, with *β*-phellandrene having an *IP* of 6.32 eV, an *EA* of 0.55 eV, and an *ω* of 2.05 eV, while *α*-phellandrene has an *IP* of 6.04 eV, an *EA* of 0.57 eV, and an *ω* of 2.00 eV. Both compounds exhibit good antioxidant potential and controlled reactivity. *ρ*-cymene [54], with an *IP* of 6.59 eV, an *EA* of 0.18 eV, and an *ω* of 1.79 eV, ranks next, showing balanced properties [57]. Sabinene, caryophyllene, and *l*-limonene present moderate antioxidant potential, with sabinene having an *IP* of 6.21 eV, an *EA* of −0.29 eV, and an *ω* of −1.35 eV, caryophyllene having an *IP* of 6.25 eV, an *EA* of −0.22 eV, and an *ω* of 1.40 eV, and *l*-limonene having an *IP* of 6.49 eV, an *EA* of −0.36 eV, and an *ω* of 1.37. *α*-pinene ranks the lowest, with an *IP* of 6.37 eV, an *EA* of −0.53 eV, and an *ω* of 1.23 eV, indicating it is less favorable for antioxidant properties compared to the other compounds [58]. This analysis highlights *β*-myrcene as the best candidate for antioxidant and anti-cell proliferation properties, followed by *β*-phellandrene, *α*-phellandrene, *ρ*-cymene, sabinene, caryophyllene, *l*-limonene, and *α*-pinene.

**Figure 2:**
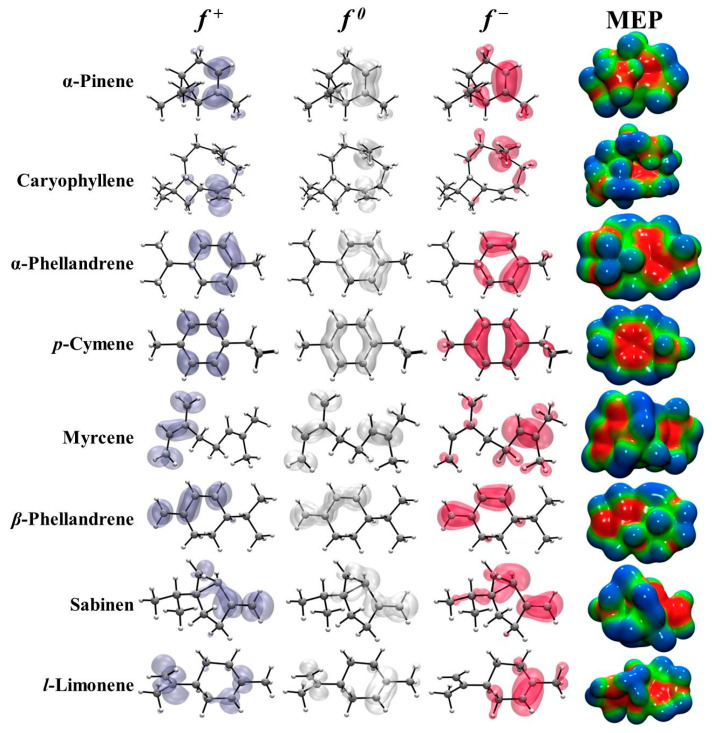
Isosurface representation of the electrophilic *f*^−^, radical *f*^0^, and nucleophilic *f*^+^ Fukui functions and molecular electrostatic potential maps generated with electron density for SM_EO molecules. Electrostatic potentials are mapped on the surface of the electron density of 0.01 a.u. The red surface corresponds to a negative region of the electrostatic potential (−0.001 a.u.), whereas the blue color corresponds to the region where the potential is positive (0.009 a.u.).

The calculation of local reactivity indices via the Koopman approach enabled us to concurrently present the electrophilic and/or nucleophilic activation at various molecular sites. For this analysis, the Fukui functions [32,59] were utilized. These functions can be interpreted as measures of how the chemical potential responds to changes in the external potential or how the electron density fluctuates as the electron count in the system varies. Furthermore, they facilitate the prediction of reactive sites susceptible to nucleophilic, electrophilic, and radical attacks. The isosurface plots of Fukui functions (see Figure 2) provide valuable insights into the reactive sites of the SM_EO molecules and their susceptibility to different types of chemical attacks. The Fukui function for electrophilic attack (*f*^−^) highlights regions prone to accepting electrons, with higher values (shown in red) indicating sites more susceptible to electrophilic attacks. Conversely, the Fukui function for nucleophilic attack (*f*^+^) identifies regions prone to donating electrons, where higher values (shown in blue) signify sites more vulnerable to nucleophilic attacks. Furthermore, the Fukui function for radical attack (*f*^0^) reveals regions susceptible to both accepting and donating electrons, with higher values (shown in white) indicating sites more prone to radical attacks. These isosurfaces serve as visual representations of the reactive sites of the SM_EO compounds. This allows us to predict and understand the reactivity of these molecules to various chemical reactions, such as electrophilic, nucleophilic, and radical attacks. In the case of *α*-pinene, caryophyllene, *α*-phellandrene, *ρ*-cymene, *β*-phellandrene, sabinene, and *l*-limonene, the isosurfaces of *f*^−^, *f*^+^, and *f*^0^ are present in their molecular rings or cycles and in their hydrocarbon chains. In the case of *β*-myrcene, the isosurfaces of *f*^−^, *f*^+^, and *f*^0^ are present only in its hydrocarbon chains. Considering the potential application of *β*-myrcene as an antioxidant, it is interesting to know the active sites for radical attack.

The distributions of electrons within SM_EO molecules are represented by molecular electrostatic potential (MEP) maps, as depicted in Figure 2. MEPs enable the prediction of physicochemical properties and provide information regarding the nature and shape of electrostatic forces, whether positive, negative, or neutral. Based on this electrostatic distribution, MEPs can estimate the favorability of electrophilic or nucleophilic attacks during interactions with other molecules or radicals. The positive regions (blue/green colors) indicate the portions of the molecule most likely to undergo nucleophilic attack. These are the electron-deficient regions that can accept electrons from a nucleophile, whereas the negative regions (red/yellow colors) indicate regions that will undergo electrophilic attack. These are the electron-rich regions that can donate electrons to an electrophile. The MEP map for SM_EO molecules represents the distribution of electrostatic potential around the molecules using color coding on an electron density isosurface. The regions exhibiting the most negative electrostatic potential (red color) are situated around the carbon atoms, as carbon is more electronegative than hydrogen, leading to a higher electron density. These regions indicate the most favorable sites for electrophilic attack or interaction with electron-deficient species. Conversely, regions displaying the most positive electrostatic potential (blue color) are located around the hydrogen atoms, which possess a partial positive charge due to their electronegativity difference with carbon. These regions signify the most favorable sites for nucleophilic attack or interaction with electron-rich species.

#### 3.2.2. *In Silico* Pharmacokinetic Prediction

For a compound to be used in preparations, in addition to showing the desired effect, it is necessary for it to show adequate distribution in tissues and to be harmless. For SM_EO, consisting of at least 25 different compounds and with 8 of these molecules accounting for 95.4% of the total, it would be interesting to evaluate the biochemical, pharmacokinetic, and safety behavior of each of its major components. For this purpose, the absorption, distribution, metabolism, excretion, and toxicity (ADMET) profiles were estimated to identify whether they possess properties within a critical range for the success of a drug candidate [60,61,62,63], as well as their toxicity.

**Figure 3:**
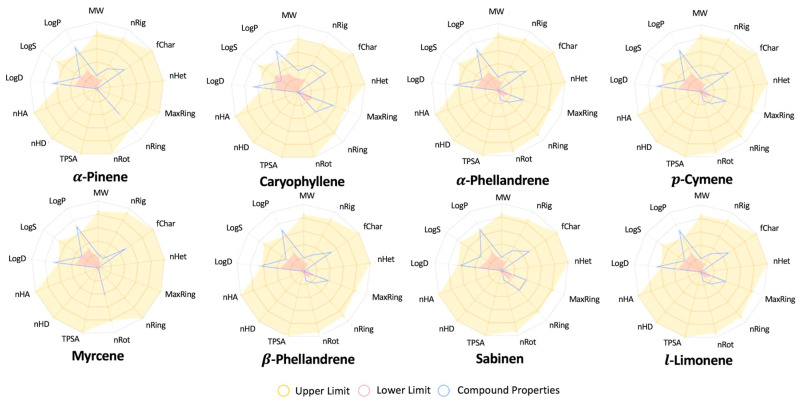
Physicochemical property analysis using bioavailability radar plot representations for SM_EO molecules. The shaded area represents the range of properties to be considered drug-like. The blue line represents the properties of the test molecules. The radar plot angles represent the following: molecular weight (MW), logarithm of n-octanol/water (LogP), logarithm of aqueous solubility (LogS), logarithm of n-octanol/water at pH = 7.4 (LogD), number of hydrogen bond acceptors (nHA), number of hydrogen bond donors (nHD), topological polar surface area (TPSA), number of rotatable bonds (nRot), number of rings (nRing), number of atoms in the biggest ring (MaxRing), number of heteroatoms (nHet), formal charge (fChar), and number of rigid bonds (nRig).

The radar plot (Figure 3) shows that sabinene exhibits physicochemical properties within the appropriate range. For the other compounds, the moderately elevated LogP/LogD values (2.1–4.3) suggest enhanced membrane permeability but potential tissue accumulation—a characteristic shared by many successful plant-derived drugs like artemisinin (LogP ~3.5) and taxol (LogP ~3.7) [64]. While this lipophilicity may require formulation optimization (e.g., nanoemulsions [65], it falls within the safe range observed for FDA-approved terpenoids, such as limonene (LogP 4.5, GRAS status).

**Figure 4:**
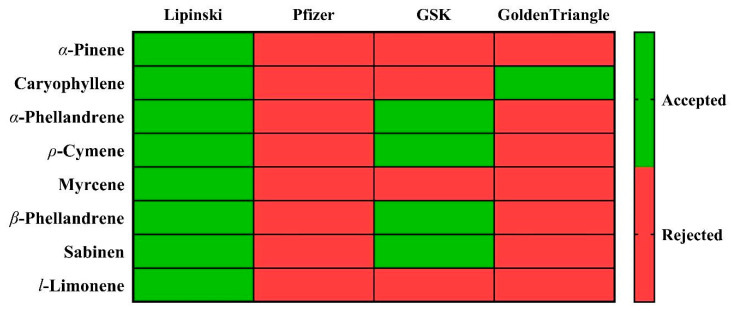
Heatmap of the Lipinski, Pfizer, GlaxoSmithKline (GSK), and Golden Triangle rules offers a comprehensive assessment of the drug likeness of SM_EO molecules. Each cell in the heatmap corresponds to a specific compound and indicates its adherence to the respective rules. Green cells signify full compliance with the rules, indicating acceptance, while red cells denote non-compliance with any of the rules, signaling rejection for drug-likeness analysis.

An interesting area of *in silico* analysis is determining whether the physicochemical properties of the compounds analyzed are similar to drugs already in commercial use. Thus, an analysis was performed using four drug selection criteria: the “Lipinski, Pfizer, GlaxoSmithKline (GSK), and Golden Triangle” rules. The minor deviations from ideal drug-likeness parameters mirror those of clinically successful phytochemicals like curcumin [60], suggesting these borderline properties do not necessarily preclude therapeutic potential but may require targeted delivery strategies. Notably, the observed CYP inhibition alerts should be interpreted cautiously, as volatile terpenes frequently show false positives *in silico* [61], and they are widely used for this purpose (Figure 4).

Lipinski’s rule [60] considers that the molar mass should be less than 500 g/mol, with a reduced hydrophobicity (LogP ≤ 5) and a moderate number of hydrogen acceptors (Hacc ≤ 10) and donors (Hdon ≤ 5). This criterion indicates that if two of the indices exceed the ranges, the absorption and permeability could be unacceptable, which is noted in red in Figure 4. The Pfizer rule considers inadequate a LogP value greater than 3 and a topological polar surface area (TPSA) value less than 75, with absorption and permeability being evaluated as poor when one of the criteria is not met (red for the Pfizer column in Figure 4). Compliance with GlaxoSmithKline (SGK) rules estimates whether a molecule shows a favorable ADMET profile, considering MW less than or equal to 400 and LogP less than or equal to 4. This index evaluates as excellent (green) those values that comply with both criteria and poor otherwise (red). Finally, the “Golden Triangle” rule was evaluated, which considers 200 ≤ MW ≤ 50; −2 ≤ LogD ≤ 5, the rule being satisfied for compounds meeting all the criteria.

As can be seen in the figure, all the analyzed compounds meet the Lipinski criterion. However, only *α*-phellandrene, *ρ*-cymene, *β*-phellandrene, and sabinene meet the GSK criterion. In contrast, only caryophyllene meets the Golden Triangle criterion.

**Figure 5:**
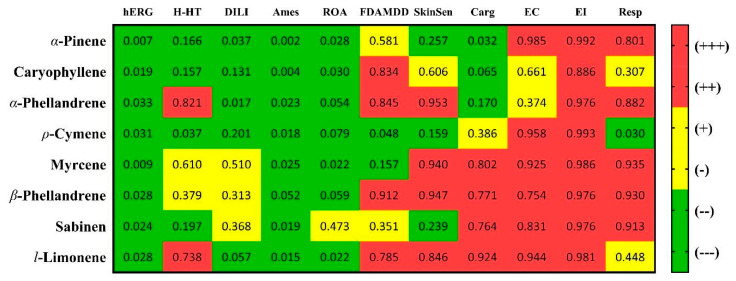
Heatmap illustrating the main toxicological indices of SM_EO molecules, providing a comprehensive overview of their potential safety profiles. Each cell in the heatmap corresponds to a specific compound and displays prediction probability values categorized into six intervals: 0–0.1 (−−−), 0.1–0.3 (−−), 0.3–0.5 (−), 0.5–0.7 (+), 0.7–0.9 (++), and 0.9–1.0 (+++). These values signify the likelihood of toxicity, with lower probabilities indicating a lower risk (−−−) and higher probabilities suggesting a higher risk (+++). To facilitate interpretation, the heatmap utilizes a color scheme: green represents the lowest prediction probability; yellow indicates medium-to-normal prediction probability; and red highlights the highest prediction probability.

To estimate the toxicity level of the main components of SM_EO, the following indices were used: human ether-a-go-go-related gene (hERG), human hepatotoxicity (H-HT), drug-induced liver injury (DILI), the Ames test for mutagenicity (Ames), and rat oral acute toxicity (ROA). The maximum recommended daily dose provides an estimate of the toxic dose threshold of chemicals in humans (FDAMDD) [skin sensitization (SkinSen), carcinogenicity (Carg), eye corrosion (EC), eye irritation (EI), and respiratory toxicity (Resp)]. Each of these indices addresses an aspect that makes it possible to evaluate the level of toxicity of the compounds. As shown in Figure 5, all compounds show values below the limit for the hERG, H-HT, DILI, Ames, and ROA indices, except for α-phellandrene and l-limonene, which exceed the H-HT index. In the case of EI, all of the compounds studied exceed the maximum tolerable value. In general, the compounds meeting the highest number of criteria are caryophyllene, *α*-phellandrene, and *ρ*-cymene.

### 3.3. Antioxidant Activity

To measure the antioxidant potential of SM_EO, five complementary *in vitro* assays were used to determine the free radical scavenging and trapping capacity. The results of the antioxidant activity are presented in Table 2.

The DPPH and ABTS assays were used to measure the free radical scavenging potential of SM_EO. The free radical scavenging activity of SM_EO was evaluated by incubating the oil with the radical DPPH and ABTS; the reduction of radicals was accompanied by a color change from violet to yellow and from blue-green to yellow, respectively. These results show that, for the inhibitory concentrations (IC_50_) of the free radicals present in solution, determined graphically and expressed as μg/mL, SM_EO presented a better capacity to capture the DPPH radical, with an IC_50_ of the order of 48.89 ± 0.82 μg/mL, while for the ABTS radical, the inhibition was lower, with an IC_50_ of 62.07 ± 1.25 μg/mL, respectively. In both cases, Trolox showed better antioxidant activity than the EO.

The FRAP assay was used to quantify the capacity of SM_EO to reduce the ions from Fe^3+^ to Fe^2+^ in solution in the presence of TPTZ as a complexing agent. The result showed a value of 21.45 ± 1.56 mg Eq Trolox/g_EO_ for the SM_EO. The chelation of ferrous ions induced by SM_EO was estimated using the method of ferrozine as a chelating reagent that forms complexes with the free ferric ion (Fe^3+^) but not with the ferrous ion (Fe^2+^) present in the reaction mixture. In the presence of chelating agents such as EDTA or compounds present in EO, the formation of ferrous complexes and ferrozine is altered, leading to a decrease in the color of the complex. Therefore, this decrease in color allows the metal chelating activity to be estimated. SM_EO decreased dose-dependently the absorbance of the Fe^2+^-ferrozine complex, with IC_50_ values of 145.06 ± 1.54 μg/mL. EDTA showed very strong activity (IC_50_ 12 ± 0.18 μg/mL) compared to SM_EO.

### 3.4. Antibacterial Activity

Antimicrobial activity was evaluated via the agar diffusion method, and MIC was determined via microdilutions (see Table 3). The highest antimicrobial effect was observed in the clinical isolate of *B. cereus*, with a MIC value equivalent to 2.0 μg/μL. The ATCC strains showed sensitivity to SM_EO, with *Y. enterocolitica* ATCC 9338361, *S. enteritidis* ATCC 13076, and *S. typhimurium* ATCC 14,028 exhibiting a MIC of 32.5 μg/μL, as well as the clinical isolates of *S. flexneri* and *S. sonnei*. The monoterpenes *α*-phellandrene and limonene (purchased from Sigma-Aldrich^®^, accordingly) tended not to overcome the effect of essential oil. Strong inhibition results were obtained from the clinical isolates *S. flexneri* for *α*-phellandrene (MIC of 8.5 μg/μL) and *S. boydii* for limonene (MIC of 16.8 μg/μL).

### 3.5. Cytotoxicity and Biological Activity

**Figure 6:**
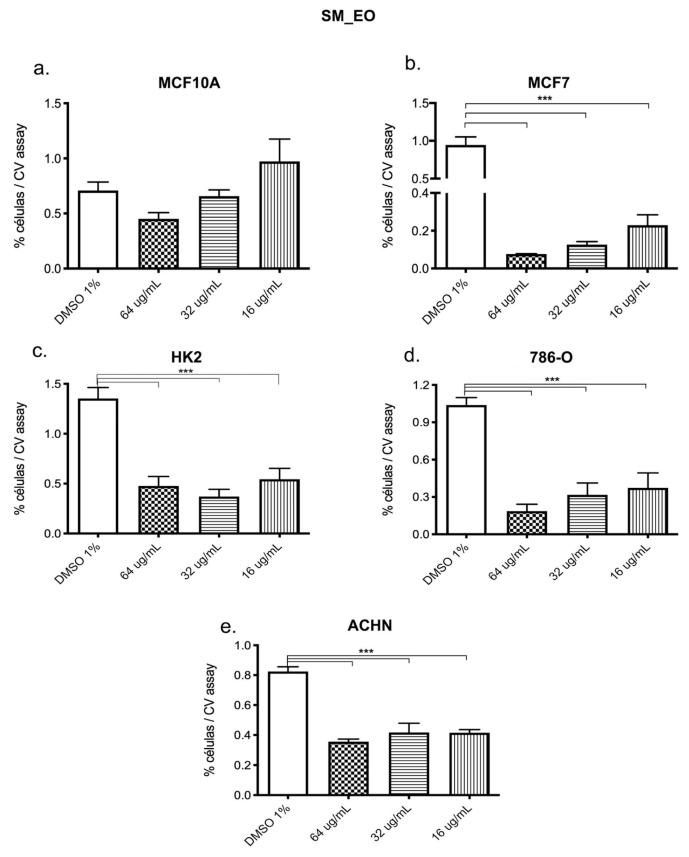
Proliferation assay of human epithelial tumor and non-tumor cells from: mama breast MCF10A (**a**) and MCF7 (**b**) and kidney 786-O (**d**), ACHN (**e**), and HK-2 (**c**) treated with vehicle (DMSO 1%) or SM_EO at different concentrations for 48 h, evaluated by via CV assay at 570 nm. The graph bar represents the mean +SEM of the values by obtained triplicate of from three independent experiments performed in triplicate independent per each treatment, the *** represent a *p* < 0.001. In the MCF10A cell line (**a**), the *p* value was 0.062.

To understand whether SM_EO treatment modified molecules related to cellular functions like survival and proliferation and whether the potential effect was selective of the “status” of the cell (non-tumor versus tumor stage), we analyzed the survival percentage of cells treated in a dose-dependent manner with decreasing concentrations of SM_EO versus the vehicle (DMSO 1%). Using the crystal violet proliferation assay, we found that in the human epithelial non-tumoral mammary MCF10A cells treated with SM_EO, the viability was not modified significantly compared with the vehicle (*p* = 0.062) (Figure 6a). However, when the tumoral mammary cell line MCF7 was treated with the SM_EO at different concentrations, selective cytotoxicity was significantly evident with respect to the vehicle (*p* < 0.001) (Figure 6b). With respect to the human renal epithelial cells, we found viability to be decreased in both non-tumoral (HK-2) and tumoral cells (786-O and ACHN) with respect to the vehicle (DMSO 1%), with this difference reaching statistical significance in all cases (*p* < 0.001) (Figure 6c–e). We observed similar results when the SM_EO effect was measured previously via the MTT assay.

**Figure 7:**
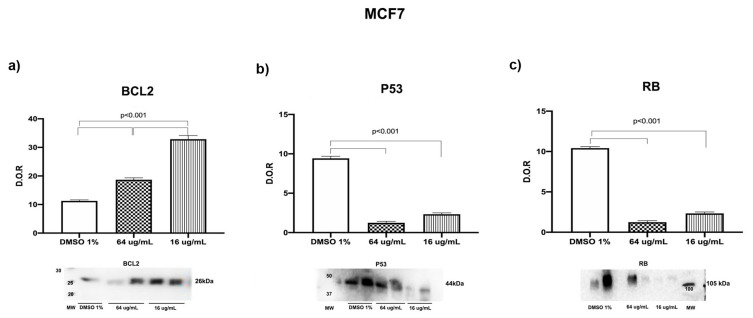
Cell cycle proteins expression of MCF7 cells treated with SM_EOs at different concentrations. Western blot iThe images of the western blot shown the present presence and abundance of BCL-2 (**a**), P53 (**b**), and RB (**c**) proteins on in the cells treated with vehicle (DMSO 1% or SM_EOs) for 48 h, evaluated by via CV assay at 570 nm. The graph bar represents the mean +SEM of the values, quantified by Fiji Image J software (x86-64), by obtained from three independent experiments performed in triplicate of three experiments independent per each treatment.

To understand the potential mechanisms or pathways involved in the proliferation and viability decrease, we employed Western blot in order to analyze the abundance of proteins related to cell cycle functions like proliferation and survival in the human epithelial mammary tumor MCF7 cells treated with 64 and 16 ug/mL SM_EO compared with the vehicle (DMSO 1%). We chose this cell line because the cytotoxicity effect was significantly evident with respect to the vehicle (Figure 7). We found that MCF7 cells treated with SM_EO showed a significant increase in BCL-2 in a dose-dependent manner (which was more evident when cells were treated with 16 ug/mL SM_EO) and a significant decrease in P53 and RB proteins (*p* < 0.001) (Figure 7).

### 3.6. Toxicity

**Figure 8:**
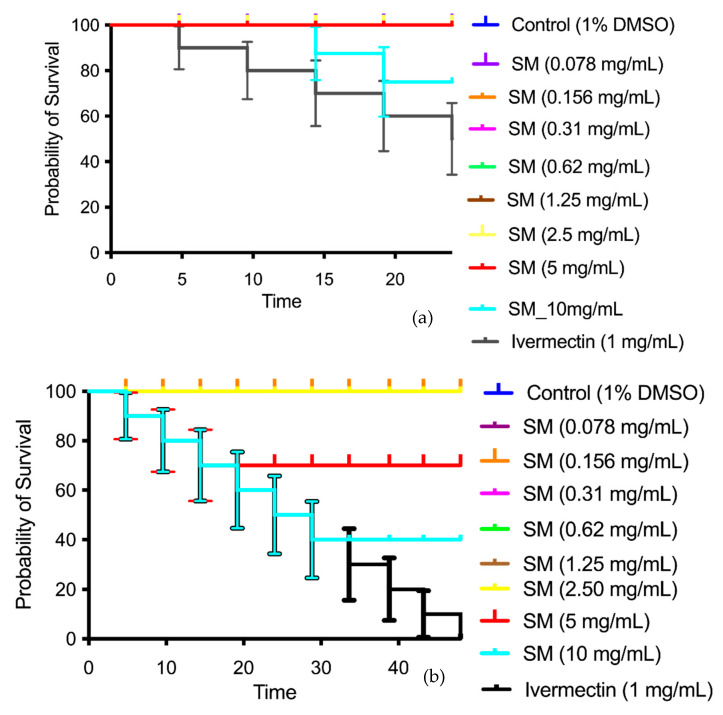
Percent survival of *Caenorhabditis elegans* exposed to SM_EO for 24 h (**a**) and 48 h (**b**).

SM_EO showed low toxicity against *Caenorhabditis elegans* in the concentration range of 0.078–10 mg·mL^−1^ (Figure 8); however, increased toxicity was noted at the concentration of 10 mg·mL^−1^ at 24 h of exposure (Figure 8a). At 48 h (Figure 8b), the toxicity increased at concentrations of 5–10 mg·mL^−1^. Ivermectin (nematicide) was used as a lethality control (positive control), killing 100% of the worms at a concentration of 0.3 mg·mL^−1^.

## 4. Discussion

Previous studies have reported that the SM_EO is composed mainly of monoterpene hydrocarbons and some sesquiterpenes. Their major constituents are α-phellandrene, β-phellandrene, α-pinene, β-pinene, β-myrcene, limonene, β-elemene, copane, germacrene, γ-cadinene, and α-humulene in fruits and leaves. These are attributed to antibacterial, antifungal, anti-inflammatory, cytotoxic, insecticidal, phytotoxic, and antioxidant activities [8,9,10,22,23,25,66,67]. The chemical composition can vary depending on geographical location and extraction methods [68]. The chemical characterization of SM_EO from the Atacama Desert through GC-MS analysis allowed us to recognize a characteristic chemotype of monoterpenes, whose relative abundances were directly correlated with soil potassium and phosphorus contents and inversely correlated with soil acidity and its lime, nitrogen, and organic carbon contents. The chemical composition of an EO varies depending on the climatic conditions to which the plant is exposed, as well as the extractive techniques [69]. This makes its chemical composition rich in various secondary metabolites, which have varied biological activities [70]. Overall, the presence of terpenes, terpene derivatives, and non-terpene compounds stands out, with these being responsible for the medicinal activity of essential oils. The different biological activities described for SM_EO could be related to the synergism of this mixture of terpenes, as well as the effect exerted by non-volatile compounds [71]. SM_EO presented a high concentration of β-phellandrene (7.01%), limonene (13.99%), β-myrcene (23.87%), and α-phellandrene (34.02%), all derived from 1,4 cyclohexadiene, highlighting that the presence of conjugated double bonds is especially useful because π bonds are responsible for the chain-breaking antioxidant activity in monoterpenes [72].

In this study, we conducted an *in silico* analysis using quantum chemistry calculations and ADME-Tox predictions to evaluate the properties of the individual components of SM_EO. This approach allowed us to obtain precise predictions of the reactivity of the compounds and their potential use as new drugs. In this way, we could quickly estimate the individual activity of each compound, addressing the problem in a manner similar to previous studies [21,73]. The *in silico* study supports this claim, highlighting the α- and β-phellandrene compounds for their high reactivity based on the energy gap and HOMO–LUMO energy orbitals. According to the reactivity descriptor calculations, β-myrcene could be an excellent antioxidant agent, a characteristic also possessed by α- and β-phellandrene, though to a lesser extent. Furthermore, the compounds that follow as candidates for antioxidant and antiproliferative activities are α-phellandrene, β-phellandrene, α-cymene, sabinene, caryophyllene, l-limonene, and α-pinene. However, the predicted reactivity of these molecules would not cause toxicity based on the analysis of their physicochemical properties. The predictions indicate that all analyzed compounds meet the safety criteria based on their physicochemical properties, with the exception of a slight deviation in LogP and LogD values. Additionally, they comply with the Lipinski and toxicological criteria. These analyses indicate the individual contribution of each component to the observed effect in the mixture (Figure 3). These analyses also demonstrate a consistently higher activity via methods involving electron transfer mechanisms, such as FRAP, while the activity shown in assays involving hydrogen transfer, such as DPPH and ABTS, is lower. The large presence of monoterpenes in the oil, which are less soluble in water, could be a factor in the lower capacity to capture free radicals in the ABTS test with respect to the activity shown with the DPPH radical, which uses an alcoholic matrix (Table 2). The SM_EOs have been studied for their chemical composition and antioxidant activities. GC-MS analyses revealed that the EOs are rich in monoterpene hydrocarbons, particularly α-phellandrene, β-phellandrene, and limonene [10,22]. The antioxidant activity of SM_EO was evaluated using various methods (*in vitro*), including DPPH, ABTS, and FRAP assays (Table 2). Generally, the SM_EO showed low-to-moderate antioxidant activity, with better performance in assays involving electron transfer mechanisms like FRAP compared to hydrogen transfer methods like DPPH and ABTS [22,66]. The antioxidant capacity was found to be influenced by chemical composition, with heavier fractions containing higher concentrations of compounds like terpinen-4-ol and germacrene D exhibiting greater antiradical power [74].

Antimicrobial activity was assessed using agar diffusion (Table 3) and MIC via microdilutions (Table 3). The clinical isolate of *Bacillus cereus* exhibited the highest antimicrobial effect. ATCC strains, including *Yersinia enterocolitica*, *Salmonella enteritidis*, and *Salmonella typhimurium*, showed sensitivity to SM_EO, as did the clinical isolate of *Shigella flexneri*. The monoterpenes α-phellandrene and limonene did not overcome the essential oil effect, although α-phellandrene and limonene showed strong inhibition against the clinical isolates of *Shigella flexneri* and *Shigella boydii*, respectively, and limonene exhibited antimicrobial activity against *Staphylococcus aureus* [75]. Furthermore, the presence of this compound in essential oils, along with other bioactives, such as β-myrcene, has been shown to exhibit better antimicrobial properties [76]. It is important to mention that the structure of limonene has a chiral center, and it is an optically active compound with two enantiomers, (R)- and (S)-limonene, which can be found as isomers or as a mixture [77]. R-limonene is considered to be the most abundant compound in citrus. It occurs naturally and is the most active compound, and most biological activities are attributed to it [78]. In this study, given the chromatographic conditions used, it was not possible to recognize the presence of an isomer or a mixture of both; therefore, it is suggested that future studies use chiral columns that allow separation optimization of this compound and thus attribute the different properties of SM_EO to the specific enantiomer. Finally, α-phellandrene, β-phellandrene, and β-myrcene demonstrated potent antibacterial effects against *Staphylococcus aureus*, *Escherichia coli,* and *Pseudomonas aeruginosa* [10,79]. The oil also exhibited antimicrobial properties against a wide range of other bacterial species, including *Klebsiella pneumoniae*, *Proteus vulgaris*, and *Bacillus subtilis* [80].

In order to evaluate the selective cytotoxicity and antitumoral activity of the SM_EO, the mammary tumor cell line MCF7 and the non-tumor cell line MCF10A were incubated with SM_EO at different concentrations, and a proliferation CV assay was performed [81]. SM_EO significantly inhibited the proliferation of mammary tumor MCF7 cells at concentrations of 64 and 16 µg/mL. Conversely, in the MCF10A non-tumoral cells, no significant differences were found among the treatments, irrespective of the concentration (Figure 6).

We found that MCF7 epithelial cells showed an inhibited proliferation when treated with SM_EO at different concentrations (64 and 16 µg/mL). Minor concentrations of 8 µg/mL did not show any effects. The non-tumoral mammary epithelial cell line MCF10A showed a tendency to decrease in viability compared with the DMSO 1% group; however, this difference was not significant (*p* value 0.062). Meanwhile, the tumoral cell line MCF7 showed significant differences at different SM_EO concentrations. The antitumoral effect was maintained in the other tumor epithelial cell lines. Additionally, we employed Western blot in order to analyze the abundance of BCL-2, P53, and RB proteins on MCF7 cell lines treated with SM_EO. A significant increase in BCL-2 was noted, as well as significant decreases in P53 and RB proteins. This could indicate that SM_EO exposure changed the molecular expression of proteins related to some of the cancer hallmarks, and these were correlated with a minor proliferation and survival of epithelial tumor cells (MCF7). However, future studies are necessary to explore the possible mechanism responsible for this effect exerted by SM_EO.

Human epithelial renal cells (HK-2, 786-O, and ACHN) exposed to SM_EO were analyzed. In contrast to the effect observed in non-tumoral mammary cells, SM_EO showed an inhibitory effect on the proliferation of the non-tumor renal cell line HK-2 (Figure 6c). Regarding the primary tumor (786-O) and metastatic site (ACHN) renal cell lines, treatment with SM_EO demonstrated a significant inhibition of cell viability and proliferation (Figure 6d–e, respectively). The SM_EO showed a greater inhibitory effect on these epithelial tumor cell lines. Limonene, a monoterpene found in citrus fruits, exhibits significant anticancer potential across various cancer types, particularly in lung cancer. Studies have shown that limonene inhibits cancer cell growth and suppresses tumor development in animal models [82,83]. Its anticancer mechanisms involve inducing apoptosis and autophagy in cancer cells, upregulating pro-apoptotic genes like BAX, and activating caspase pathways [82,84]. Limonene also modulates key signaling pathways, including Ras/Raf/MEK/ERK and PI3K/Akt, while increasing P53 expression and decreasing VEGF levels [84]. In hepatocellular carcinoma cells, limonene significantly altered the expression of numerous cancer-related genes, affecting processes such as apoptosis induction, signal transduction, and cell cycle regulation [85]. These findings highlight limonene’s potential as a promising, cost-effective anticancer agent warranting further investigation. Using Western blot, we observed an overexpression of BCL-2 when cells were exposed to SM_EO (Figure 7). Previous authors demonstrated that an increase in BCL-2 can reduce oxygen free radical production and lipid peroxide formation. BCL-2 can reduce the transmembrane flow of calcium ions, suggesting that BCL-2 regulates apoptosis through calcium channels. Apoptotic factors accumulate on the endoplasmic reticulum and release Ca^2+^ [86], activating the precursor caspase 12, which in turn activates caspase 9 and caspase 3 and finally leads to apoptosis. The above three pathways finally converge to the same pathway; that is, activated caspase 8, caspase 9, and caspase 12 all cut and activate caspase 3, which ultimately leads to apoptosis [86]. On the other hand, we observed a significant decrease in the P53 and RB proteins, and these were correlated with a decrease in the proliferation of epithelial tumor cells from breast and kidney. It has been reported that BCL-2 is a mitochondrial protein endowed with cytostatic and anti-apoptotic properties [87]. Our results suggest that changes in the expressions of BCL-2 and P53 when cells are exposed to SM_EO affect the distribution of cells among the cell cycle phases and modify the sensitivity to cytotoxic drugs and the type of cell death. Regarding RB, we found this protein to be significantly decreased with SM_EO treatment. A previous work showed that one downstream consequence of RB activity is inhibition of E2F activity, which is important for the transcription of several genes that are required for progression through the G1 and S phases of the cell cycle. RB also regulates P53 activity through a trimeric P53–MDM2–RB complex [87]. Future studies are necessary to understand the mechanism underlying this activity; this will generate added value to the study of the mechanisms of activity of the chemical components of SM_EO and its potential applications in biomedicine.

*C. elegans* is an interesting model for evaluating the toxicity of compounds using a complete animal model with processes conserved in mammals [88]. We studied the possible toxicity of SM_EO in *C. elegans* and observed that it presented low lethality at concentrations of 0.078–10 mg·mL^−1^ when evaluated via assays at 24 and 48 h (Figure 8a,b). Research on the majority components in EO revealed concentration-dependent effects on nematode survival, with reproduction being a more sensitive endpoint than lethality [89]. Silica particles functionalized with majority components in the EO demonstrated varying toxicological effects, with vanillin-functionalized particles generally showing milder acute toxicity [89]. *C. elegans* has also been used to assess the biosafety of nanoparticles, providing insights into the sublethal effects on development, intestinal function, immune response, neuronal function, and reproduction [64]. These studies highlight the potential of *C. elegans* as a complete model organism for toxicity evaluations, offering a cost-effective and ethically sound alternative to mammalian models. Similar findings have been reported in other studies on EOs extracted from Chilean herbs, demonstrating toxicity only at higher concentrations (50 or 25 mg/mL), thus confirming that these EOs present low toxicity [21,48,52].

## 5. Conclusions

The comprehensive chemical characterization of SM_EO from the Atacama Desert highlights a complex mixture, predominantly composed of monoterpenes and some sesquiterpenes, with α-phellandrene, β-myrcene, limonene, and β-phellandrene being the major constituents. These compounds play a central role in conferring the wide range of biological activities observed in SM_EO, including strong antioxidant, antibacterial, cytotoxic, and toxicity-modulating effects.

Based on the *in silico* analysis, α- and β-phellandrene exhibit the highest nucleophilic reactivity, positioning them as promising candidates for antioxidant and antiproliferative applications. β-myrcene stands out as a particularly potent antioxidant, further supporting the therapeutic potential of the SM_EO blend. The combined presence of these compounds may lead to synergistic effects, enhancing the mixture’s overall efficacy against oxidative stress and cellular proliferation.

Moreover, ADME-Tox predictions suggest that these molecules do not pose significant toxicity risks and demonstrate favorable pharmacokinetic properties. All compounds comply with Lipinski’s Rule of Five and exhibit suitable physicochemical characteristics, reinforcing their suitability for future drug development. These findings support the potential of SM_EO as a safe and effective source of bioactive compounds.

The results also highlight the strong antimicrobial potential of SM_EO, particularly attributed to the high reactivity of monoterpenes such as α-phellandrene and limonene, which showed marked inhibition of clinically relevant and pathogenic bacterial strains. The synergistic action of diverse secondary metabolites further enhances the bioactive profile of the oil, reinforcing its promise for a range of medicinal applications.

Given the observed selective antiproliferative effect on MCF7 cancer cells, low in vivo toxicity in *C. elegans,* and acceptable *in silico* pharmacokinetic profiles, SM_EO emerges as a promising candidate for future development in pharmacological, cosmetic, or sanitation applications. Nevertheless, further studies are needed to explore formulation strategies and validate its efficacy in complex biological systems.

Future studies should focus on isolating and optimizing the most active constituents to enhance their therapeutic precision and applicability. Additionally, combining computational and experimental approaches will deepen the understanding of the molecular mechanisms underlying these bioactivities and support the translation of these findings into practical applications. These may include pharmaceutical treatments for cancer and inflammation, as well as natural antimicrobial solutions for veterinary use.

## Figures and Tables

**Table 1:** Results of GC-MS analysis of *Schinus molle* (SM) essential oil (EO).

		Composition					
N°	tr (min)	R.I. ^e^	R.I. ^t^	Area (%)	Compounds	Id	CAS
**1**	3.545	930	931	4.80	α-pinene	GC-MS, R.I.	80-56-8
**2**	3.873	942	946	0.16	camphene	GC-MS, R.I.	79-92-5
**3**	4.586	967	969	3.52	sabinene	GC-MS, R.I.	3387-41-5
**4**	**5.279**	**991**	**991**	**23.87**	**β-myrcene**	GC-MS, R.I.	**123-35-3**
**5**	**5.557**	**1001**	**1000**	**34.02**	**α-phellandrene**	GC-MS, R.I.	**99-83-2**
**6**	6.236	1025	1026	5.36	p-cymene	GC-MS, R.I.	99-87-6
**7**	**6.305**	**1028**	**1027**	**7.01**	**β-phellandrene**	GC-MS, R.I.	**555-10-2**
**8**	**6.320**	**1028**	**1028**	**13.99**	**limonene ^1^**	GC-MS, R.I.	
**9**	7.246	1061	1062	0.06	γ-terpinene	GC-MS, R.I.	99-85-4
**10**	8.034	1089	1088	0.14	terpinolene	GC-MS, R.I.	586-62-9
**11**	9.075	1132	1129	0.41	octanoic acid, methyl ester	GC-MS, R.I.	111-11-5
**12**	9.498	1151	1145	0.22	limonene oxide, cis	GC-MS, R.I.	13837-75-7
**13**	10.111	1178	1177	0.05	terpinen-4-ol	GC-MS, R.I.	562-74-3
**14**	10.681	1204	1202	0.19	α-phellandrene epoxide	GC-MS, R.I.	288393-04-4
**15**	13.798	1377	1377	0.04	α-copaene	GC-MS, R.I.	3856-25-5
**16**	14.102	1394	1387	0.13	(E)-β-elemene	GC-MS, R.I.	33880-83-0
**17**	14.371	1410	1410	0.42	1H-cycloprop[e]azulene, 1a,2,3,4,4a,5,6,7b-octahydro-1,1,4,7-tetramethyl	GC-MS, R.I.	489-40-7
**18**	14.502	1418	1418	1.77	β-caryophyllene	GC-MS, R.I.	87-44-5
**19**	15.054	1454	1455	0.27	α-caryophyllene	GC-MS, R.I.	6753-98-6
**20**	15.178	1462	1462	0.09	(Z,E)-α-farnesene	GC-MS, R.I.	502-61-4
**21**	15.494	1482	IRNR	0.17	α-amorphene	GC-MS, R.I.	483-75-0
**22**	15.752	1499	1498	0.64	bicyclogermacrene	GC-MS, R.I.	67650-90-2
**23**	15.841	1504	1504	0.07	α-muurolene	GC-MS, R.I.	10208-80-7
**24**	16.184	1527	1523	0.34	δ-cadinene	GC-MS, R.I.	483-76-1
**25**	16.976	1579	1577	0.10	caryophyllene oxide	GC-MS, R.I.	1139-30-6

^1^ This compound does not have a CAS number because it presents chirality and may represent a single isomer, a racemic mixture, or an enantiomeric excess. e: experimental (retention or relative Kovats index calculated relative to a C10-C30 n-alkane standard on an Rtx-5MS capillary column); t: theoretical (retention or relative Kovats index reported in the literature). The compounds with the greatest relative abundance in the SM_EO are indicated in bold.

**Table 2:** SM_EO. Activities for various antioxidant systems.

Oil	IC_50_ ABTS ^a^	IC_50_ DPPH ^a^	IC_50_ Ferrozine ^a^	FRAP ^b^
**SM_EO**	62.07 ± 1.25	48.89 ± 0.82	145.06 ± 1.54	21.45 ± 1.56
**Trolox**	26.48 ± 1.60	27 ± 0.51	----	----
**EDTA**	----	----	12 ± 0.18	----

All values are expressed as means ± SEM (*n* = 4). **^a^** expressed in µg/mL. **^b^** expressed in mg Trolox equivalent/mL of SM_EO.

**Table 3:** MICs of SM_EO and its monoterpenes against bacteria (clinical isolates and ATCC).

Microbial Strain	SM_EO MIC (μg·μL^−1^)	α-Phellandrene MIC (μg·μL^−1^)	Limonene MIC (μg·μL^−1^)
*B. cereus* *	2.0	34	67.4
*S. flexneri* *	32.5	8.5	33.7
*Y. enterocolitica*	32.5	34	33.7
*S. enteritidis*	32.5	68	67.4
*S. sonnei* *	32.5	68	67.4
*S. typhimurium*	32.5	68	67.4
*C. striatum* *	65	-	-
*E. coli*	65	68	67.4
*E. faecalis*	65	136	-
*S. epidermidis*	65	136	134.7
*S. paratyphi* *	65	68	134.7
*S. sciuri*	65	68	67.4
*L. monocytogenes*	130	-	134.7
*S. aureus*	130	68	-
*S. boydii* *	130	-	16.8

* C.I: Clinical isolates. (-) No inhibition observed at the highest concentration tested. ANOVA followed by Tukey’s test did not show significant differences between the purified compounds of α-phellandrene and limonene. The values represent the average of clinical isolates (obtained from the Chilean population). The experiment was performed in triplicate.

## Data Availability

The original contributions presented in this study are included in the article/Supplementary Materials. Further inquiries can be directed to the corresponding author.

## Supplementary Materials

The following supporting information can be downloaded at: https://www.mdpi.com/article/10.3390/plants14152449/s1, Table S1: Summary of equations used to calculate various global reactivity indexes in the TAFF pipeline; Table S2: Global reactivity indexes in eV, for *α*-pinene, caryophyllene, *α*-phellandrene, *ρ*-cymene, *β*-myrcene, *β*-phellandrene, sabinene, and *l*-limonene.

## Abbreviations

EO, essential oil; MC, majority compound; AC, antioxidant capacity; GC-MS, gas chromatography–mass spectrometry; MIC, minimum inhibitory concentration; HD, hydrodistillation; SM, *Schinus molle*; MW, molecular weight; TPSA, topological polar surface area; HBA, H-bond acceptor; HBD, H-bond donor; RB, distribution of the number of rotatable bonds; Log P, decimal logarithm of the partition coefficient between n-octanol and water; Log S, decimal logarithm of the molar solubility in water; Log Kp, prediction of the skin permeability coefficient; FRAP, ferric-reducing antioxidant power; HOMO, highest occupied molecular orbital; LUMO, lowest unoccupied molecular orbital; IP, ionization potential; EA, electron affinity; χ, electronegativity; η, global hardness; ω, electrophilicity index; ω−, electro-donating; ω+, electro-accepting; Δω±, electrophilicity net; f+, f0, and f−, Fukui functions; MEP, molecular electrostatic potential; ADMET, absorption, distribution, metabolism, excretion, and toxicity; LogP, logarithm of n-octanol/water; LogS, logarithm of aqueous solubility; LogD, logarithm of n-octanol/water at pH = 7.4; nHA, number of hydrogen bond acceptors; nHD, number of hydrogen bond donors; nRot, number of rotatable bonds; nRing, number of rings; MaxRing, number of atoms in the biggest ring; nHet, number of heteroatoms; fChar, formal charge; nRig, number of rigid bonds; SGK, GlaxoSmithKline; hERG, human ether-a-go-go-related gene; H-HT, human hepatotoxicity; DILI, drug-induced liver injury; Ames, the Ames test for mutagenicity; ROA, rat oral acute toxicity.
