# Supplementary material for: Exploring the Bioactive Potential and Chemical Profile of Schinus molle Essential Oil: An Integrated In Silico and In Vitro Evaluation
Source: Plants (Basel). 2025 Aug 7;14(15):2449. doi: 10.3390/plants14152449 (PMC12349550; doi:10.3390/plants14152449)
Supplement: Supplementary file 1 [file plants-14-02449-s001.zip › plants-3750936-supplementary.pdf]

Table S1. Summary of equations used to calculate various global reactivity indexes in the TAFF pipeline

	Koopmans' theorem	*Reference*
Ionization potential (*IP*)	$I = -\epsilon_H$	[38, 39]
Electron affinity (*EA*)	$A = -\epsilon_L$	[38, 39]
Global Hardness (η)	$\eta = \frac{1}{2}(\epsilon_L - \epsilon_H)$	[38, 39]
Electronegativity (χ)	$\chi = -\frac{1}{2}(\epsilon_L + \epsilon_H)$	[40-43]
Electrophilicity (ω)	$\omega = \frac{\mu^2}{2\eta} = \frac{(\epsilon_L + \epsilon_H)^2}{2(\epsilon_L - \epsilon_H)}$	[44]
Electron Acceptor (ω^+)	$\omega^+ = \frac{(\epsilon_L + 3\epsilon_H)^2}{16(\epsilon_L - \epsilon_H)}$	[44]
Electron Donator (ω^-)	$\omega^- = \frac{(3\epsilon_L + \epsilon_H)^2}{16(\epsilon_L - \epsilon_H)}$	[44]
Net Electrophilicity ($\Delta\omega^\pm$)	$\Delta\omega^\pm = \omega^+ + \omega^-$	[45]

These indexes are calculated using CDFT descriptors derived from the HOMO (ϵ_H) and LUMO (ϵ_L) energies.

Table S2. Global reactivity indexes in eV, for *α*-pinene, caryophyllene, *α*-phellandrene, *ρ*-cymene, *β*-myrcene, *β*-phellandrene, sabinene, and *l*-limonene

SM_EO	ϵ_H	ϵ_L	GAP	*IP*	*EA*	η	ω	χ	ω^+	ω^-	$\Delta\omega^\pm$
α-pinene	−6.37	0.53	6.91	6.37	−0.53	3.45	2.92	1.23	0.21	3.12	3.33
caryophyllene	−6.25	0.22	6.48	6.25	−0.22	3.24	3.01	1.4	0.3	3.31	3.61
α-phellandrene	−6.04	−0.57	5.46	6.04	0.57	2.73	3.3	2.00	0.69	3.99	4.68
ρ-cymene	−6.59	−0.18	6.41	6.59	0.18	3.21	3.39	1.79	0.50	3.89	4.39
β-myrcene	−6.32	−0.68	5.64	6.32	0.68	2.82	3.50	2.18	0.78	4.28	5.06
β-phellandrene	−6.32	−0.55	5.77	6.32	0.55	2.88	3.44	2.05	0.69	4.13	4.82
sabinene	−6.21	0.29	6.51	6.21	−0.29	3.25	2.96	1.35	0.27	3.24	3.51
l-limonene	−6.49	0.36	6.85	6.49	−0.36	3.43	3.06	1.37	0.27	3.33	3.59

Note Table S2. The chemical reactivity descriptors presented here are calculated using Koopmans' approximation based on HOMO (Highest Occupied Molecular Orbital) and LUMO (Lowest Unoccupied Molecular Orbital) energies. Ionization Potential (IP) represents the energy required to remove an electron, while Electron Affinity (EA) corresponds to the energy released upon gaining an electron. Electronegativity (χ) is calculated as the average of IP and EA, and global hardness (η) as half their difference, indicating resistance to changes in electron density. The electrophilicity index (ω) quantifies electrophilic capacity, while the electroaccepting (ω^+) and electrodonating (ω^-) powers describe specific electron transfer capabilities. Finally, net electrophilicity ($\Delta\omega\pm$) integrates these effects to assess overall reactive behavior. The highlighted values for β-myrcene (high ω, ω^-, and $\Delta\omega\pm$) reveal its strong tendency to participate in electron transfer processes, making it the most reactive component in the analyzed group.
